# Supplementary material for: Testosterone regulates CYP2J19-linked carotenoid signal expression in male red-backed fairywrens (Malurus melanocephalus)
Source: Proc Biol Sci. 2020 Sep 16;287(1935):20201687. doi: 10.1098/rspb.2020.1687 (PMC7542802; doi:10.1098/rspb.2020.1687)
Supplement: Supplementary methods and tables for “Testosterone regulates CYP2J19-linked carotenoid signal expression in male red-backed fairywrens (Malurus melanocephalus)” [file rspb20201687supp1.docx]

**Testosterone regulates CYP2J19-linked carotenoid signal expression in male red-backed fairywrens (*Malurus melanocephalus*)**

Sarah Khalil, Joseph F. Welklin, Kevin J. McGraw, Jordan Boersma, Hubert Schwabl, Michael S. Webster, Jordan Karubian

Proceedings of the Royal Society B

DOI: 10.1098/rspb.2020.1687

**Supplementary Methods:**

Testing for differences in GAPDH Ct between phenotypes

To test that GAPDH expression, which we used as our housekeeping gene for our delta-delta CT analysis (below), did not differ between phenotypes, we ran a linear mixed effect model with the lme function in the R package nlme, R v. 3.6.0. The model included GAPDH Ct as the response variable and phenotype (female vs. unornamented male vs. vs. testosterone implanted unornamented male vs. ornamented male) as the predictor variable. In addition, we added individual sample as a random effect to control for repeated measures since each sample was run in triplicate during qPCR. We tested the model for significance with an ANOVA using the aov function in R, and found no significant effect of phenotype on GAPDH Ct (Table S3, Figure S2).

In addition, we calculated the intra-assay coefficient of variance (CV) for the GAPDH and CYP2J19 qpcr assays and found they were 1.13% and 2.13% respectively. Generally, intra-assay %CV should be below 10 to assume sufficient repeatability of the assay.

Analyzing qPCR data with delta-delta CT method

We analysed qPCR data using the delta-delta Ct method [S1], which reports gene expression (mRNA abundance) for the gene of interest as the fold change in expression (2^-ΔΔCt^), normalized to a housekeeping gene and calibrated to a “calibrator sample”. We use GAPDH as our housekeeping gene, as it is often used as a housekeeping gene for qPCR analysis in birds [S2–S5], and confirmed GAPDH Ct did not differ between treatments (above). We averaged the ΔCt (Ct CYP2J19 – Ct GAPDH) for all three females and used that average as our calibrator sample. We present our results as log fold change. Using the average female ΔCt value as our calibrator sets the average log fold change value for females at zero, allowing for easier visualization of the difference in relative gene expression between phenotypes. We also ran this analysis with only one female as our calibrator sample, and our statistical results remain exactly the same since these are all relative expression levels.

Assessing the effect of the presence of absence of a sham implant

In order to assess whether or not having a sham implant affected expression levels for those unornamented males, we combined the two sham-implanted males and the unmanipulated unornamented male into one phenotype category we called “control unornamented male.” We ran a linear model using the lm function in R with CYP2J19 expression (log fold change) as the response variable, and presence of an implant (yes vs. no) nested within phenotype (female vs. control unornamented male vs. testosterone-implanted unornamented male vs. ornamented male) as the predictor variable. We found no interaction between phenotype and implant, and the implant variable did not improve the fit of the model, suggesting there was no significant effect of not having the sham implant within our control unornamented males. However, we acknowledge that due to the small sample size, the absence of a statistical effect of presence or absence of the implant may not be substantial. In addition, we evaluated raw data and confirmed that the log fold change for the unmanipulated male (2.20) was similar to that of the two sham implant males (1.95 and 2.19, and can be seen in figure S3), and the variance of the log fold change for control unornamented males (all three males) was very small, as can be seen in the standard error bars in Figure 2. Taken all together, we therefore dropped the implant variable from our model.

Testing for an effect of sex on CYP2J19 expression

To test for a general effect of sex on liver CYP2J19 expression, we ran a linear model using the lm function in R. The model included CYP2J19 expression (log fold change) as the response variable, and sex as well as testosterone treatment nested within sex as the predictor variables - lm(logfoldchange ~ sex + sex/treatment), where sex was either male or female, and treatment was either yes or no. Only males had the option to have a “yes” treatment (the unornamented testosterone-implanted males).

There was an overall sex effect, but no significant sex*treatment interaction effect, which we believe is because the treatment birds had expression levels in between the control unornamented males and the ornamented males, therefore hiding the treatment effect when one just looks at all males together in comparison to females. The full results of the ANOVA on the linear model can be found in Table S4.

**Supplementary Tables and Figures:**

|  | | | | |  |  |  |
| --- | --- | --- | --- | --- | --- | --- | --- |
|  | Value | Std Error | DF | t-value | | p-value | |
|  | | | | | | |  |
| (Intercept) | 16.128 | 1.422 | 84 | 11.338 | | ﻿<0.001* | |
| Phenotype (Unornamented Male) | -26.030 | 2.761 | 34 | -9.429 | | ﻿<0.001* | |
| Phenotype (Female) | -14.378 | 1.499 | 84 | -9.594 | | ﻿<0.001* | |
| Age | -0.161 | 0.407 | 34 | -0.396 | | 0.695 | |
| Phenotype (Unornamented Male)*Age | 13.489 | 2.220 | 34 | 6.076 | | ﻿<0.001* | |
| Phenotype (Female)*Age | 0.410 | 0.438 | 34 | 0.936 | | 0.356 | |
|  | | | | | | |  |

**Table S1:** The effect of the fixed predictor variables on circulating metabolized carotenoid concentration using a linear-mixed model, with individual as a random effect. This model includes the 123 samples of birds with exact known age. Results are qualitatively similar to the model with all 160 samples (Table S2).

|  | | | | |  |  |  |
| --- | --- | --- | --- | --- | --- | --- | --- |
|  | Value | Std Error | DF | t-value | | p-value | |
|  | | | | | | |  |
| (Intercept) | 15.062 | 1.146 | 105 | 13.139 | | ﻿<0.001* | |
| Phenotype (Unornamented Male) | -24.959 | 2.366 | 49 | -10.548 | | ﻿<0.001* | |
| Phenotype (Female) | -12.971 | 1.229 | 105 | -10.557 | | ﻿<0.001* | |
| Age | 0.027 | 0.344 | 49 | 0.079 | | 0.937 | |
| Phenotype (Unornamented Male)*Age | 13.310 | 1.908 | 49 | 6.975 | | ﻿<0.001* | |
| Phenotype (Female)*Age | 0.119 | 0.375 | 49 | 0.318 | | 0.752 | |
|  | | | | | | |  |

**Table S2:** The effect of the fixed predictor variables on circulating metabolized carotenoid concentration using a linear-mixed model, with individual as a random effect. This model includes all 160 samples.

|  | | | | |  |  |  |
| --- | --- | --- | --- | --- | --- | --- | --- |
|  | Value | Std Error | DF | t-value | | p-value | |
|  | | | | | | |  |
| (Intercept) | 26.608 | 0.408 | 24 | 65.278 | | ﻿<0.001* | |
| Phenotype (Unornamented Male) | -0.309 | 0.576 | 8 | 0.536 | | ﻿0.6067 | |
| Phenotype (Female) | 0.307 | 0.576 | 8 | 0.533 | | ﻿0.6086 | |
| Phenotype (T-implanted nornamented male) | 0.0263 | 0.576 | 8 | 0.456 | | 0.6603 | |
|  | | | | | | |  |

**Table S3:** The effect of the fixed predictor variable (phenotype) on GAPDH Ct using a linear-mixed model, with sample as a random effect.

|  | Df | Sum Sq | Mean Sq | F-value | p-value | |
| --- | --- | --- | --- | --- | --- | --- |
|  | | | | | |  |
| Sex | 1 | 14.7120 | 14.7120 | 148.3946 | ﻿<0.001* | |
| Sex:treatment | 1 | 0.3954 | 0.3954 | 3.9882 | 0.07692 | |
| Residuals | 9 | 0.8923 | 0.0991 |  |  | |
|  | | | | | |  |

**Table S4:** Analysis of variance table for sex-effect linear model: lm(logfoldchange ~ sex + sex/treatment)


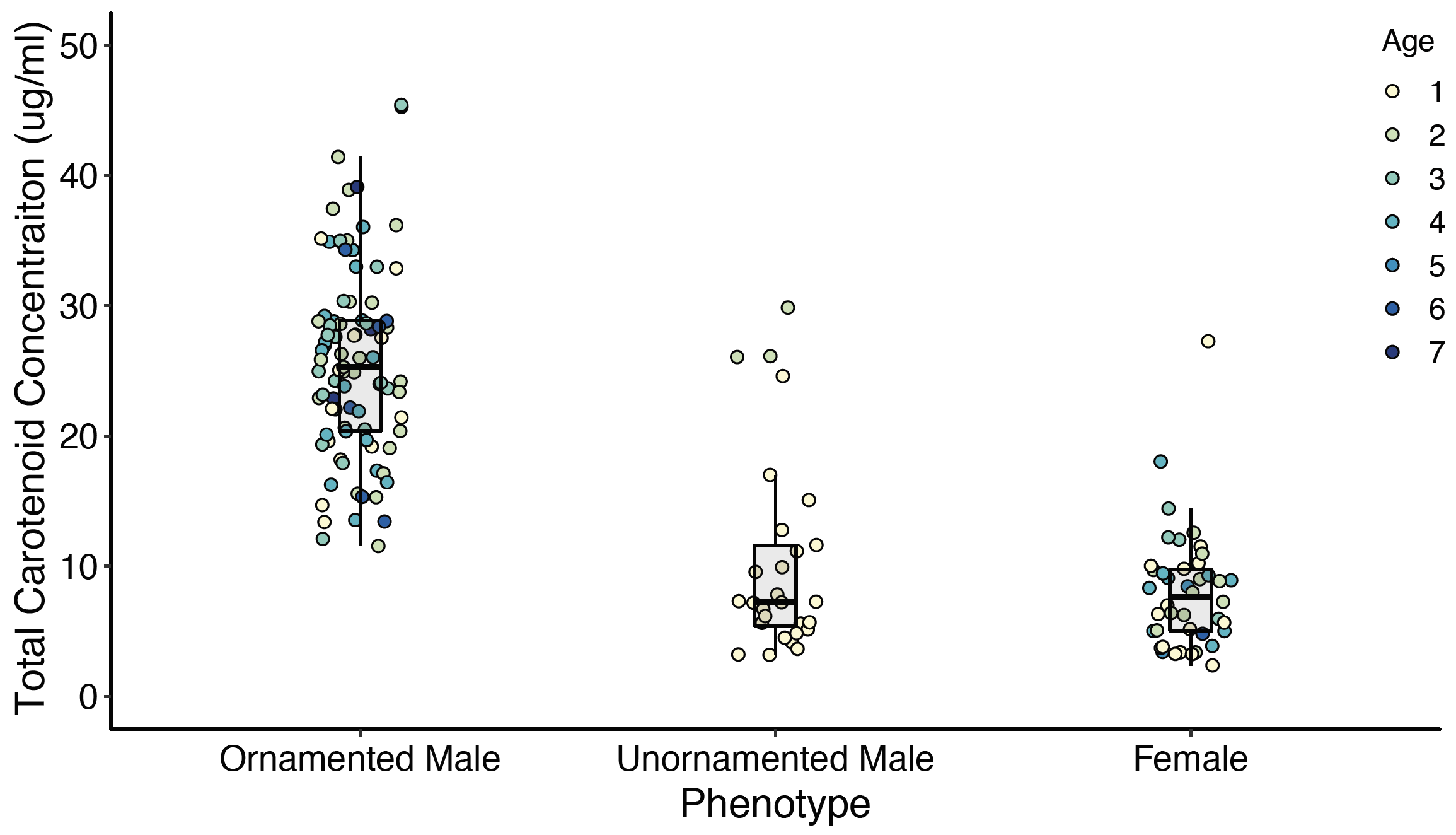


**Figure S1 -** Total circulating carotenoid concentration for the different phenotypes are presented as boxplots, indicating the median and quartiles with whiskers reaching up to 1.5 times the interquartile range. The scatterplot points represent the value of the total carotenoid concentration for each sample and the colour of each point represents the minimum age of the bird in that sample (see inset legend). Note that within unornamented males, 2-year old males had higher concentrations of circulating carotenoids than 1-year old males.


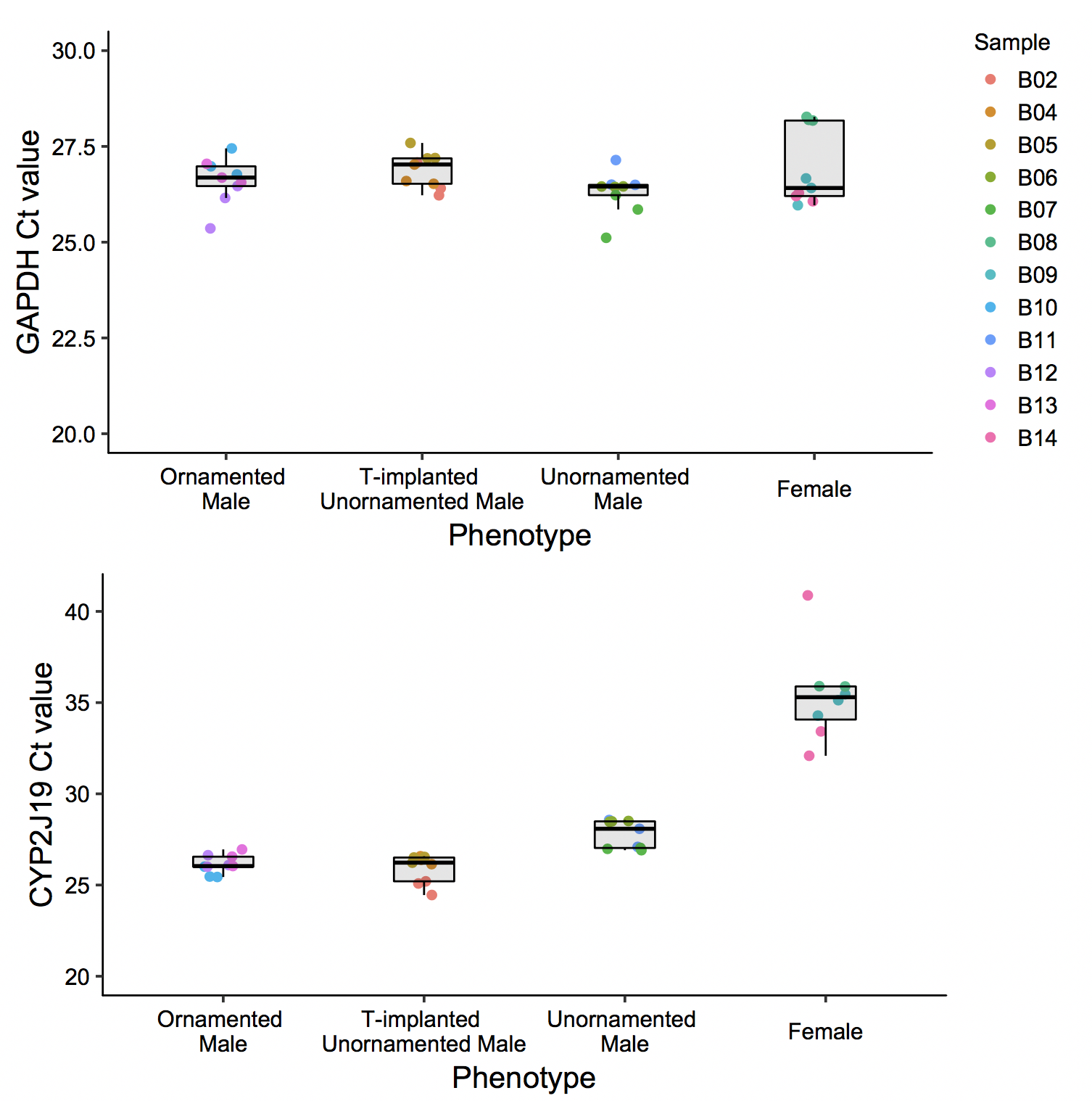


**Figure S2** – Ct values for GAPDH do not differ between phenotypes. Shown are Ct values for (A) GAPDH and (B) CYP2J19 for ornamented males, testosterone-implanted unornamented males, control unornamented males, and females. Points are coloured by sample, as each sample was run in triplicate for qPCR, except for one female sample for CYP2J19 which was run in duplicate (B08). Higher Ct values are reflective of less mRNA in the sample.


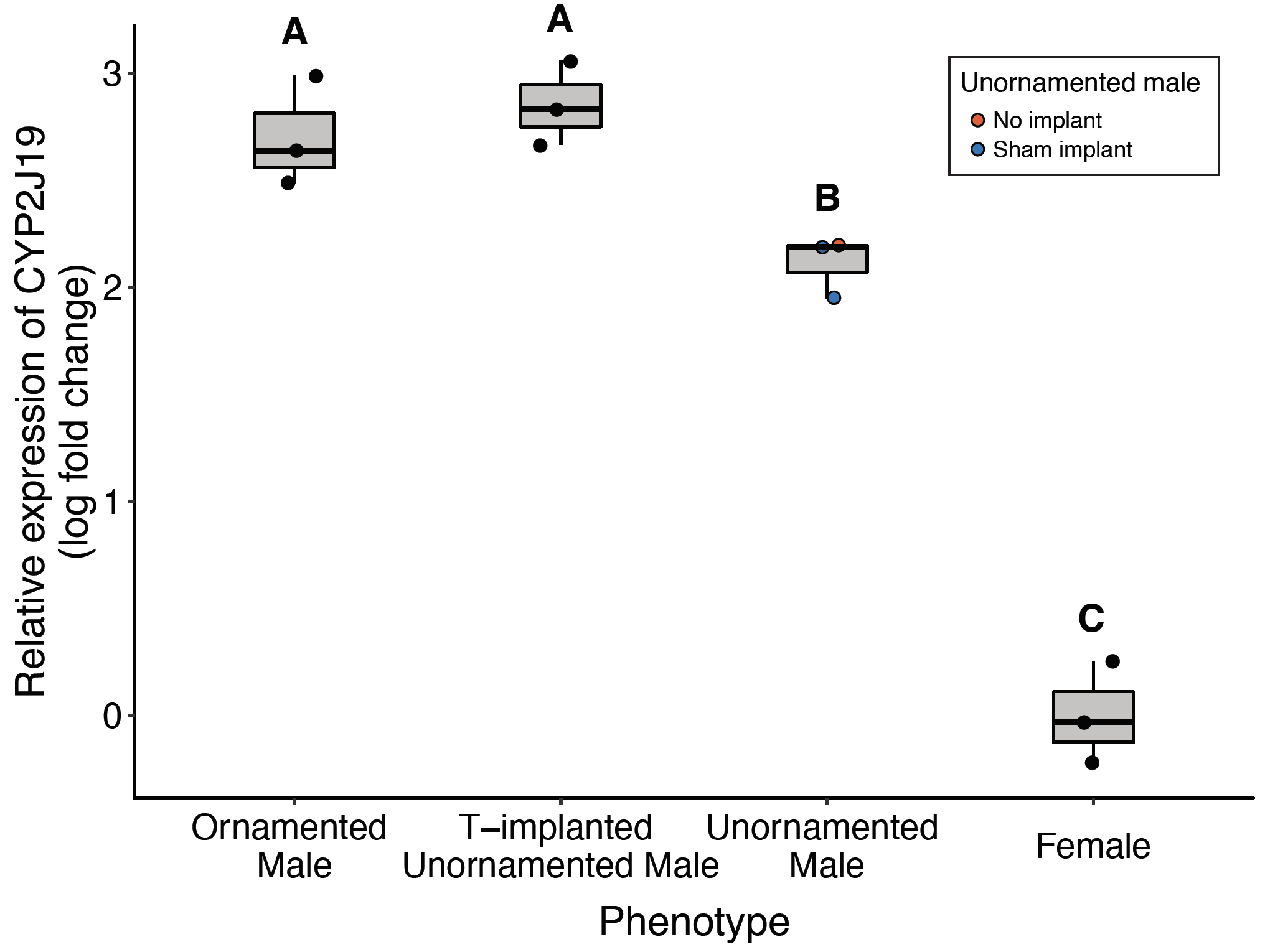


**Figure S3 –** Liver CYP2J19 expression of sham-implanted unornamented male is similar to that of non-implanted unornamented male. Shown are qPCR measurements of expression of CYP2J19 relative to housekeeping gene GAPDH in ornamented males, testosterone-implanted unornamented males, control unornamented males, and females of the red-backed fairywren (this is the same as Figure 2). Points represent samples from individual birds, and boxplots indicate the median and quartiles with whiskers reaching up to 1.5 times the interquartile range. Different letters above plots indicate significant differences at p<0.05 by Tukey’s HSD. Colour of points for the “unornamented male” group represent individuals who either had a sham-implant (blue), or the one individual who did not have any implant (orange).

**Supplementary References:**

S1. Livak KJ, Schmittgen TD. 2001 Analysis of Relative Gene Expression Data Using Real-Time Quantitative PCR and the 2−ΔΔCT Method. *Methods* **25**, 402–408. (doi:10.1006/meth.2001.1262)

S2. Toomey MB *et al.* 2017 High-density lipoprotein receptor SCARB1 is required for carotenoid coloration in birds. *Proc. Natl. Acad. Sci.* **114**, 5219–5224. (doi:10.1073/pnas.1700751114)

S3. Lopes RJ *et al.* 2016 Genetic Basis for Red Coloration in Birds. *Curr. Biol.* **26**, 1427–1434. (doi:10.1016/j.cub.2016.03.076)

S4. Twyman H, Prager M, Mundy NI, Andersson S. 2018 Expression of a carotenoid-modifying gene and evolution of red coloration in weaverbirds (Ploceidae). *Mol. Ecol.* **27**, 449–458. (doi:10.1111/mec.14451)

S5. Gazda MA, Toomey MB, Araújo PM, Lopes RJ, Afonso S, Myers CA. 2020 A Genetic Mechanism for Sexual Dichromatism in Birds. *Science.* **12**, 1–6. (doi:10.1126/science.aba0803)
